# Sustainability of integrated hypertension and diabetes with HIV care for people living with HIV at primary health care in South Ethiopia: implication for integration

**DOI:** 10.1186/s12875-023-02204-4

**Published:** 2023-11-17

**Authors:** Abebe Sorsa Badacho, Ozayr Haroon Mahomed

**Affiliations:** 1 School of Public Health, Wolaita Sodo University, PO 138, Wolaita Sodo, Ethiopia; 2 School of Nursing and Public Health, Public Health Medicine Discipline, University of KwaZulu-Natal, Durban, South Africa; 3 Health Economics and HIV and AIDS Research Division (HEARD), University of KwaZulu-Natal, Durban, South Africa; 4 Dasman Diabetes Institute, Kuwait City, Kuwait

**Keywords:** Sustainability, Integration, PLWH, Hypertension, Diabetes

## Abstract

**Background:**

People living with human immunodeficiency virus (PLWH) have an increased risk of developing noncommunicable diseases (NCDs) compared with people without HIV. The multimorbidity of NCDs and HIV increases the need for integrated care. However, there is a paucity of evidence on the implementation of integrated sustained hypertension and diabetes with HIV care to address the multiple chronic care needs of PLWH in Ethiopia.

**Objective:**

This study aimed to determine the sustainability of integrated hypertension and diabetes within HIV care for PLWH in primary healthcare (PHC) in southern Ethiopia.

**Methods:**

The National Health Service Institute for Innovation and Improvement Sustainability Model (NHS- SM) self-assessment tool was used to assess sustainability. HIV care and NCD team members from five PHC facilities in South Ethiopia were included. Participants completed the self-administered NHS-SM assessment tool independently.

**Result:**

The overall mean sustainability was 43.74 (95% CI: 42.15–45.33). All facilities had an overall sustainability score of less than 55. The perceived benefit beyond helping the patient, the likelihood of adaptability, and perceived alignment with the organizational goal were identified as potential factors promoting sustainability. The perceived lack of an effective system to monitor progress, staff behavior, inadequate staff involvement and training, inadequate senior leadership support and clinical leadership engagement, and infrastructure limitations could negatively affect sustainability.

**Conclusions:**

Integrating hypertension and diabetes with HIV care sustainably at PHC requires staff involvement and training, staff behavior change communication, ensuring PHC management and clinical leadership (doctors and senior clinicians) engagement, and addressing infrastructure limitations.

## Background

People living with human immunodeficiency virus (PLWH) have an increased risk of developing noncommunicable diseases (NCDs), especially cardiovascular disease (CVD), cervical cancer, and diabetes, compared with people without HIV [1, 2]. With increased coverage of antiretroviral therapy (ART), the life expectancy of PLWH has improved, exposing them to an increased risk of NCD [2].

The WHO recommends enhanced NCD screening, diagnosis, treatment, and control through integration with primary healthcare (PHC) [3], emphasizing how NCDs affect different groups of people, such as PLWH, and designing programs targeting health determinants [3]. The UNAIDS Global AIDS Strategy 2021–2026, End Inequalities and End AIDS, aims to achieve 90% of PLWH by 2025, which is linked to people-centered and context-specific integrated services for NCDs that are needed for overall health and well-being [4]. Integration of NCDs into HIV service delivery models and the provision of integrated care contribute to sustaining the gains made through the expansion of ART and improved access to NCD services [2].

Studies indicate that initiatives to prevent NCDs in Ethiopia are relatively cost-effective [5]; however, there are gaps in implementing cost-effective preventive and clinical interventions [5]. A national survey conducted in Ethiopia indicated that the overall availability and readiness to provide NCD-related health services are suboptimal [6]. NCD services are generally provided in hospitals, which creates multiple challenges to patient access and increases the burden of NCDs [1]. Owing to the lack of integrated HIV and NCD care in Ethiopia, PLWH do not receive routine hypertension and diabetes screening at the same HIV care clinics [7].

Evidence demonstrates that initiatives to implement new evidence-based practices usually fail to achieve long-term effects [8–11]. The literature has reported that approximately 40% of new initiatives are not sustained while the initial funding is terminated [12]. Sustainability means the continuation or integration of new practices within an organization whereby it has become a routine part of care delivery and continues to deliver desired outcomes [11, 13]. Therefore, assessing the likelihood of sustainability intervention is crucial for clients, practitioners, policy-makers, and funders [14].

There is a paucity of evidence on the empirical study of integrated hypertension and diabetes with HIV care at PHC, its sustainability, and the scalability of the intervention in Ethiopia. This study aimed to assess and inform determinants of sustainability of integrating hypertension and diabetes with HIV care at PHC that could improve intervention outcomes and wider adoption and promotion of people-centered care to address the multiple chronic care needs of PLWH.

## Materials and methods

### Study design

This was part of a larger mixed-method study. The current study was an observational cross-sectional study with an analytical component conducted from October 15 ,2022, to November 10, 2022.

### Study area

This study was conducted at five selected PHC facilities in the Wolaita Zone of southern Ethiopia, which provide comprehensive HIV care for PLWH. Only ten PHC facilities of the 68 PHC facilities in the Wolaita Zone of Southern Ethiopia provide ART services. Five ART clinics from ten PHC facilities were randomly selected. The facilities were randomly selected by listing ten PHCs providing ART services, and five PHC facilities were included using the lottery method. One PHC facility was randomly selected from each of the five districts in the Wolaita Zone of Southern Ethiopia.

### Study population

All HIV care and NCD team members involved in this study were from five selected PHC facilities (PHC- A, PHC-B, PHC-C, PHC-D, and PHC-E facilities).

### Sample size

Forty participants from HIV care and NCD care team members across five PHC facilities were selected.

### Data collection and analysis

The National Health Service (NHS) Institute for Innovation and Improvement Sustainability Model (SM) self-assessment tool was used to collect and analyze the data. The NHS-SM was designed to help teams implement new practices in their work [15]. It aims to enable teams to recognize and self-assess key variables in their local context that determine whether a new practice is likely to be sustained and prompt timely action to increase the likelihood of sustainability [15].

The NHS-SM consists of 10 factors of a comprehensive and flexible framework encompassing process (i.e., monitoring progress, adaptability, credibility of benefits, benefits beyond helping patients), staff (i.e., training and involvement, behaviors, senior leaders, clinical leaders) and organizational (i.e., infrastructure and fit with goals and culture) factors that play a vital role in sustaining change in healthcare (Table 1).

**Table 1:** Sustainability model criteria for sustainability

Description of variable	Maximum score
**Process**	**31.1**
Benefits beyond helping patients – Does the change reduce waste, duplication and added effort?	8.5
The credibility of evidence – Are the benefits to staff, patients, and organizations visible?	9.1
The adaptability of the improved process – Does the change rely on an individual, group of people or finances to keep it going?	7
Effectiveness of system to monitor progress – Is special monitoring required?	6.5
**Staff**	**52.4**
Staff involvement and training to sustain the change – Play a part in implementation and design.	11.4
Staff behavior to sustain change – Staff inputs.	11
Senior leadership engagement – Are they involved and promote it?	15
Clinical leadership engagement – Are they involved and promote it?	15
**Organization**	**16.5**
Fit with the organization’s strategic aims and culture – Is the change aligned with the organization’s strategic aims?	7.0
Infrastructure for sustainability – Staff facilities and equipment to sustain change	9.5
**Maximum score**	**100**
**Minimum sustainability score**	**55**

Although the NHS Institute for Innovation and Improvement SM tool was developed in the UK, inputs were obtained from global experts in its development. The NHS-SM tool was applied in developing countries such as South Africa and Tanzania to measure sustainability improvement initiatives [16, 17]. This study was the first to apply the NHS-SM tool to measure sustainability in an Ethiopian context. The simple nature of the tool with easy-to-understand components facilitated its application in the Ethiopian context. Sustainability is affected by numerous intrinsic and extrinsic factors and cannot necessarily be dichotomized by a single value [16]. However, to apply the SM tool, sustainability means the continuation or integration of new practices within an organization whereby it has become a routine part of care delivery and continues to deliver desired outcomes. In different contexts in the literature, a score greater than 55 indicated the sustainability of the intervention [16–19].

The primary investigator (PI) obtained a list of available HIV and NCD team members from each PHC included in the current study.

Before starting to complete the tool, the principal investigator explained how to use the NHS-SM assessment tool. NHS-SM self-assessment tools were distributed to a total of 40 members. Participants completed the self-administered NHS-SM assessment tool independently.

The data from the NHS-SM self-assessment tool were cleaned and entered into Microsoft Excel 365. The sustainability master score system was used to calculate the total score. The process, staff, and organization domain scores were summed to obtain the total sustainability score for each PHC facility. An NHS-SM master score of 55 or higher was considered optimal for sustainability. One-way ANOVA was used to determine the mean difference (p < 0.05), and the mean sustainability score was calculated. Frequency and descriptive statistics were reported.

### Data quality management

The tool was translated into the Amharic language (a language official and commonly used in Ethiopia) by the principal investigator, and then two different language experts were engaged in checking the precision and validity of the translation.

## Results

Forty participants answered the NHS-SM self-assessment tool with a response rate of 100%. Eight respondents from each of the five PHC facilities were selected for the study. Twenty-two (55%) were male, twenty-two (55%) participants had bachelor’s degrees, and seven (17%) had medical doctorate degrees. More than half of the participants (58%) had 5–10 years of PHC work experience (Table 2). Five PHC facilities serve more than 1614 PLWH for regular visits. PHC-B serves approximately 731 PLWH, and an average of 350 PLWH receive regular monthly visits from PHC.

**Table 2:** Sociodemographics of participants who answered a self-assessment tool for the sustainability of integrated hypertension and diabetes with HIV care for PLWH at PHC in southern Ethiopia

Variables		Frequency (N)	Percent (%)
Sex	Male	22	55
	Female	18	45
Age category	25–34	21	53
	35–44	15	37
	>= 45	4	10
Education status	BSc degree	22	55
	MD	7	17
	Maters	4	11
	Diploma level	7	17
year of experience category	5-10years	23	58
	11–15 years	13	32
	> 15 years	4	10
Professional category	General Physician	7	18
	Professional Nuse	7	18
	Public Health Officer	11	27
	Pharmacy	6	15
	Laboratory Technologist	5	12
	MPH	4	10

### Total sustainability scores

The overall mean sustainability of integrated hypertension and diabetes care with HIV care at the PHC level was 43.74 (95% CI: 42.15–45.33). The PHC-E had the highest mean sustainability score of 44.93 (43.37–46.49) and the lowest at PHC-A with 41.63 (95% CI: 39.77–43.5). All facilities had overall sustainability scores less than 55 (the minimum sustainability score). The mean difference in sustainability scores across the five PHC facilities was not statistically significant (*p* > 0.05).

The process component of the SM tool obtained the highest proportionate sustainability score across five PHC facilities, with a score of 17.1 ± 0.37 (mean ± SD). Only the PHC-E achieved a score greater than the minimum (17.33; 55%), with a mean process component score of 17.625 (95% CI: 14.09–21.15). The other four facilities did not achieve a minimum mean sustainability score. PHC-C had the lowest mean score of 16.57 (95% CI: 14.4 -18.74).

The staff component achieved the lowest proportionate sustainability score across five facilities, with a score of 19.75 ± 1.09 (mean ± SD). All five facilities scored below the minimum (28.87; 55%) for the sustainability of the staff component. PHC-D had the highest mean sustainability score for the staff component of 21.28 (95% CI: 18.21–24.35), and PHC-A had the lowest mean sustainability score of 18.52 (95% CI: 13.59–23.45).

The sustainability of the organizational component was 6.62 ± 0.49 (mean ± SD). The mean organizational component score of 7.33 (95% CI: -18.31–32.98) was the highest in PHC-C and the lowest in PHC-A, with a score of 6 (95% CI: -16.39–28.39). None of the facilities achieved the minimum mean organization score (9.295, 55%) (Table 3).

**Table 3:** Overall and component sustainability of integrated hypertension and diabetes with HIV care for PLWH at PHC Southern Ethiopia

Variables	PHC-A(n = 8)	PHC-B(n = 8)	PHC-C(n = 8)	PHC-D(n = 8)	PHC-E(n = 8)
Benefit	7.55(95% CI :6.07–9.02)	7.075(95% CI: 5.43–8.71)	5.38(95% CI: 3.75–7.01)	7.07(95% CI: 5.43–8.71)	7.55(95% CI: 6.079–9.02)
Credibility	3.91(95% Ci: 2.04–5.78)	4.31(95% CI: 2.34–6.28)	3.91(95% CI: 2.04–5.78)	3.9(95% CI: 2.66–5.13)	3.9 (95% CI: 2.66–5.13)
Adaptability	4.75 (95% CI: 3.19–6.30)	4.05(95% CI: 2.48–5.61)	4.95(95% CI: 3.09–6.80)	3.85(95% CI: 2.78–4.91)	3.85 (95% CI: 2.78–4.91)
Effectiveness	0.9 (95% CI: -0.13 -1.93)	1.61(95% CI: 0.46–2.75)	2.32(95% CI: 1.46–3.18)	2.325(95% CI: 1.46–3.18)	2.32(95% CI: 1.46–3.18)
**Process Domain**	17.11(95% CI: 12.75–21.46)	17.05(95% CI: 13.49–20.61)	16.57(95% CI: 14.4 -18.74)	17.15(95%CI: 13.97–20.32)	17.62(95% CI:14.09–21.15)
Staff involvement	0.61((95% CI: -0.83–2.06)	1.225(95% CI: -0.67–3.12)	1.83(95% CI: -0.28–3.95)	2.45(95% CI: 0.26–4.63)	1.837(95% CI:-0.28–3.95)
Staff behavior	3.82(95% CI: 1.85–5.79)	3.187(95% CI: 0.98–5.39)	3.18(95% CI: 0.98–5.39)	6.57(95% CI: 4.29–8.58)	6.575(95% CI: 4.29–8.85)
Senior leadership	6.6 (95% CI: 1.79–11.40)	10.537(95%: CI: 6.54–14.52)	8.21(95% CI:4.70–11.72	6.01(95% CI: 5.79–6.22)	6.07 (95% CI: 5.88–6.26)
Clinical leadership	7.48(95% CI: 3.22–11.75)	5.8 (95% CI: 5.33–6.26)	5.95 (95% CI: 5.43–6.46)	6.25(95% CI: 5.73–6.76)	5.95(95% CI: 5.43–6.46)
**Staff Domain**	18.52(95% CI: 13.59–23.45)	19.35(95% CI: 13.48–25.21)	19.18(95%CI:14.65–23.72)	21.28(95%CI: 18.21–24.35)	20.43(95% CI:16.94–23.93)
Organization aims	4.76(95% CI: 3.21–6.31)	5.22 (95% CI: 3.63–6.81)	5.68(95% CI: 4.17–7.20)	5.25(95% CI: 3.685–6.81	5.63(95% CI: 4.06–7.21)
Infrastructure	1.23(95% CI: -0.19–2.66)	1.237(95% CI: -0.19 -2.66)	1.65(95% CI: 0.175–3.12)	1.237(95% CI: -0.19–2.66)	1.23(95% CI: -0.19–2.66)
**Organization Domain**	6 (95% CI: -16.39–28.39)	6.46(95% CI: -18.87–31.79)	7.33(95% CI:-18.31- 32.98)	6.487(95% CI: -19–31.97	6.87 (95% CI: -21.07 -34.82)
**Total sustainability Score**	41.63(95% CI: 39.77–43.5)	42.86(95% CI: 40.9–44.81)	43.1(95% CI: 41.59–44.6)	44.92(95%CI:43.47–46.37)	44.93(43.37–46.49)

### Domain-level sustainability scores

The perceived benefit of integration beyond helping the patient, the likelihood adaptability of integration at the PHC level, and the perceived alignment of the integration with the organizational goal and culture were identified as the substantial factors that could promote the likelihood of sustainability of integrated hypertension and diabetes care at PHC facilities. The mean score for benefit beyond helping patients was 6.92 ± 0.89, likely hood adaptability of integration within PHC with a mean score of 4.29 ± 0.52 and the perceived alignment of integration with organizational goals and culture with a score of 5.31 ± 0.37.

Due to the perceived lack of an effective system to monitor progress, staff behaviors such as staff expressing their ideas on integration, training and practice at a small scale, perceived lack of staff involvement in design and implementation, perceived lack of senior leadership support (PHC management members) and clinical leadership engagement (involvement of doctors) and perceived lack of infrastructure were identified as factors that could negatively influence the sustainability of the integration. The actual perceived lack of infrastructure score was 1.31. There was a considerable discrepancy compared to the expected sustainability score of 9.5, which was identified as a factor that negatively influences sustainability and needs consideration when implementing integration within PHC.

**Fig. 1:**
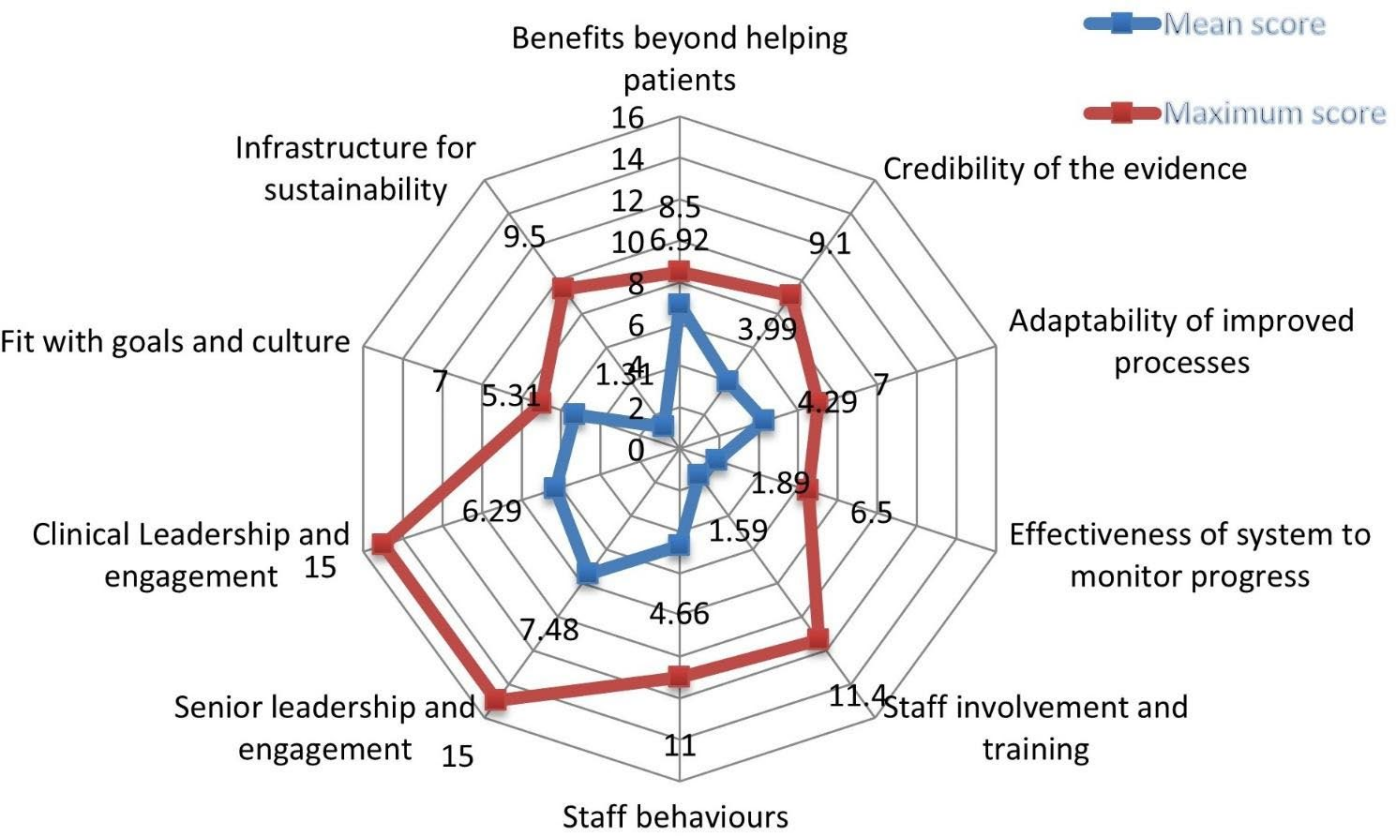
The sustainability mean component scores of integrated hypertension and diabetes with HIV are PHC facilities

The mean scores of the effectiveness of the system, staff involvement, staff behavior, senior leadership support, clinical leadership engagement and perceived lack of infrastructure were 1.89, 1.59, 4.6, 7.48, 6.28 and 1.31, respectively (Fig. 1).

## Discussion

Several studies have examined the sustainability of complex health interventions implemented in developing countries [16, 17, 20]. However, there is a paucity of evidence on the sustainability of health interventions implemented in Ethiopia. In the opinion of the researchers, this study was the first to assess the likely sustainability of integrated hypertension and diabetes with HIV care at PHC in Ethiopia; implementation has not yet started. It is crucial that this assessment was conducted to inform potential issues that should be addressed and considered in the implementation plan and could help improve the intervention implementation and scale-up and sustainability of integrated hypertension and diabetes with HIV care at PHC to address the multiple chronic care needs of PLWH.

The overall mean sustainability of integrated hypertension and diabetes with HIV care at the PHC was 43.74, ranging between 42.15 and 45.33. All PHC facilities had a lower than minimum mean sustainability score of the NHS sustainability model. This study finding was lower than that in studies performed elsewhere, such as an observational cross-sectional study conducted in South Africa; the overall mean sustainability of Integrated Chronic Care Model (ICDM) implementation at PHC was more than 55 [16]. A cross-sectional study on the sustainability of quality improvement (QI) teams in referral hospitals in Tanzania reported an overall mean sustainability score of 59.08 [17]. A volunteer-based primary care intervention in Ontario, Canada, reported a mean sustainability score of 64.6 [18]. The mean sustainability of MyDiabetesPlan at the PHC level was 69.4% in Toronto, Canada [19]. The reason for the low mean sustainability score in our study might be that the implementation of integration has not yet started. Health professionals were not exposed to implementation, so they might not perceive integration’s benefits. We examined the likelihood of sustainability of integrating hypertension and diabetes with HIV care for PLWH.

The perceived benefit of integration beyond helping the patient could be a potential factor in promoting sustainability. This finding was consistent with the perceived benefits of change felt by the teams beyond helping patients, which influenced the sustainability of QI teams in referral hospitals in Tanzania [17].

The likelihood of adaptability and the perceived alignment of the integration with the organizational goal and culture were identified as potential factors that could promote likely sustainability. This finding was consistent with the empirical study of South Africa’s perceived adaptability of ICDM implementation that can bring about organizational change reported promote sustainability [16].

Perceived inadequate staff involvement and training to play a part in integration design and implementation were found to negatively influence sustainability. Our finding was consistent with a study conducted in Ethiopia on the perceived sustainability of the school-based social and behavior change communication (SBCC) approach to malaria prevention in rural Ethiopia that reported that inadequate involvement of key stakeholders and relevant health staff and unprecedented turnover of trained staff negatively influenced sustainability [20]. The active involvement of staff in the intervention’s development, implementation, and pilot evaluation was reported as a positive factor that enhanced the sustainability of volunteer-based PHC interventions [18]. Empirical studies of the implementation of ICDM in South Africa showed that continuous staff engagement and training promoted staff knowledge, skills, and abilities and enhanced the sustainability of ICDM implementation [16]. The sustainability of QI teams in referral hospitals in Tanzania reported that perceived active involvement and training of hospital staff facilitated the sustainability of QI teams [17]. Staff involvement and training were the most significant facilitating factors for the sustainability of MyDiabetesPlan at the PHC level in the Ontario study [19]. The quality of staff training and motivation schemes could empower stakeholders for future performance [21]. The knowledge and skills of employees toward the program affect their beliefs about the sustainability of interventions [22]. A qualitative finding of the sustainability of school programs in malaria prevention in rural Ethiopia reported that the program delivered an acceptable level of critical components, such as training, supervision, review meetings, and peer education, which were found to be predictors of sustainability [20]. Training ART care providers on NCD service provision, receiving their ideas, and capacitating staff in providing integrated services could help the sustainability of the implementation of integrated services.

Perceived staff behavior of change could negatively affect integration sustainability. This finding was consistent with empirical studies of the implementation of ICDM in South Africa [16]. In contrast, the Tanzania empirical study reported that positive behavior and the readiness of QI teams supported the sustainability of quality teams, and training on concepts and practices of client care, teamwork, patient safety, and the use of various QI tools was found to be facilitators of team success and sustainability [17]. PHC management could consider encouraging and ensuring that staff can express their ideas, providing comprehensive training, and empowering them to practice small-scale implementation.

Perceived inadequate senior leadership support could adversely affect the sustainability of integration. Our finding is consistent with that of Tanzania’s empirical study, which reported that low involvement in hospital leadership (hospital management teams) adversely affected the sustainability of QI teams [17]. The empirical study of South Africa on the implementation of ICDM at PHC reported that inadequate leadership of local area managers had a negative effect on the sustainability of integration [16]. A study of the sustainability and scalability of volunteer-based PHC interventions in Ontario, Canada, reported that staff involvement and training were significant opportunities for improving sustainability [18]. The active involvement of staff in the intervention’s development, implementation, and pilot evaluation was a positive factor in enhancing sustainability [18]. A study in Toronto, Canada, indicated that inadequate senior leadership participation was a limiting factor for the sustainability and scalability of diabetic eHealth innovation implementation [19]. It is necessary to ensure that the top leaders of PHC management members are engaged, understand, and support integration within the facility, which could improve the sustainability of integration.

Perceived inadequate clinical leadership engagement and support is a critical factor that could negatively affect the sustainability of integrated services. This finding was consistent with an empirical assessment of South Africa’s implementation of ICDM, indicating that inadequate clinical leadership engagement owing to the vertical design of HIV care impacted the sustainability of integration [16]. A Tanzania study reported that inadequate clinical leaders’ (heads of clinical departments, senior nurses, and allied health workers) engagement influenced the sustainability of hospital QI teams [17]. The Ontoria study reported that clinical leadership engagement found significant opportunities for improving the sustainability and scalability of volunteer-based PHC interventions [18]. It requires effective communication to ensure that doctors are involved, understand, and promote integration.

In our study, infrastructure limitations, such as inadequate NCD equipment, such as BP cuffs, glucose meters, strips, and NCD drugs with a continuous supply, budget, and physical infrastructure, such as rooms, could negatively affect integration sustainability. This finding was consistent with a study in South Africa that reported that infrastructure limitations negatively affected the sustainability of ICDM implementation [16]. The QI teams faced increasing pressures to improve their overall performances within the limited resources allocated to implement the planned QI interventions in Tanzania [17]. Infrastructure limitations were identified as a factor that affected sustainability in the Ontario study [19]. Infrastructure limitations were reported as a significant factor that affected the sustainability and scalability of volunteer-based PHC interventions in Toronto [18]. The sustainability of healthcare teams depends, among other factors, on the availability of adequate and necessary resources and support for the team in accessing professional training and skill development [23].

### Limitations of the study

This study has numerous limitations. Our study used a self-administered sustainability assessment tool to collect data susceptible to reporting bias. We did not employ mixed methods to triangulate findings from different dimensions that could identify broad categories of factors affecting integration sustainability, such as patient-related factors. The NHS-SM has been designed for individual-planned improvement changes at a project level and does not determine the community’s or organization’s sustainability. Only HIV and NCD teams with a small sample were surveyed, not other management and staff from the facilities involved that might not be representative for generalization to other settings.

## Conclusions

The findings of this study indicated that perceived benefits beyond helping the patients, the likelihood of adaptability, and the perceived alignment of the integration with the organizational goal and culture were identified as the substantial factors that could promote the likelihood of sustainability.

The essential factors that need to be addressed for the sustainability of integration are optimal staff involvement and appropriate training of staff, staff behavior change communication, ensuring PHC management and clinical leadership (doctors and senior clinicians) engagement, and infrastructure limitations, which could negatively affect the sustainability of implementing integrated hypertension and diabetes with HIV care at PHC.

Proactive thinking in planning and carefully developing program components, such as training, stakeholder engagement, partnering, and participation, should be considered in the early design and implementation stages.

## Data Availability

The datasets generated during the current study are not publicly available because individual privacy could be compromised but are available from the corresponding author upon reasonable request.
